# Sensitivity and specificity of SARS-CoV-2 S1 subunit in COVID-19 serology assays

**DOI:** 10.1038/s41421-020-00224-3

**Published:** 2020-10-27

**Authors:** Ying Tian, Chaoyang Lian, Yingying Chen, Dong Wei, Xinxin Zhang, Yun Ling, Ying Wang, Leng-Siew Yeap

**Affiliations:** 1 Shanghai Institute of Immunology, State Key Laboratory of Oncogenes and Related Genes, Department of Immunology and Microbiology, Shanghai Jiao Tong University School of Medicine, 280 South Chongqing Road, Shanghai, 200025 China; 2 Department of Infectious Diseases, Research Laboratory of Clinical Virology, Ruijin Hospital, Shanghai Jiao Tong University School of Medicine, Shanghai, 200025 China; 3 Department of Infectious Disease, Shanghai Public Health Clinical Center, Shanghai, 201052 China

**Keywords:** Immunology, Molecular biology

Dear Editor,

Serological assays such as enzyme-linked immunosorbent assays (ELISA) using SARS-CoV-2 Spike (S) proteins are practical methods to determine the extent of COVID-19 immunity in a population upon SARS-CoV-2 vaccination^1^. However, the robustness of these assays, which depends on the sensitivity and specificity of S proteins in detecting anti-SARS-CoV-2 antibodies, is not well characterized. Here, we report that the S1 subunit of the SARS-CoV-2 S protein has superiority over the receptor-binding domain (RBD) and the native state S trimer in terms of sensitivity and specificity, respectively, in measuring anti-SARS-CoV-2 antibodies from COVID-19 convalescent patients. S1 and S trimer are more sensitive than RBD antigen because they are able to capture non-RBD, as well as RBD-binding COVID-19 antibodies. However, the full-length S trimer, which harbors S2 subunit in addition to S1, cross-reacted with antibodies elicited by circulating coronavirus (CoV), such as HCoV-OC43 and HCoV-HKU1, making it less specific than S1 in detecting COVID-19 antibodies. Our results show that the S1 subunit protein of SARS-CoV-2 is both sensitive and specific in distinguishing COVID-19 antibodies, suggesting its potential implication in large-scale serological testing, especially in determining the efficacy of SARS-CoV-2 vaccine.

CoV infection has been known to occur in humans. Some human CoV strains that are widely circulating, such as HCoV-HKU1, HCoV-OC43, HCoV-NL63, and HCoV-229E caused mild respiratory disease, while other endemic strains, such as SARS-CoV and MERS-CoV caused a more severe disease^2^. The recent outbreak of SARS-CoV-2, a virus from the *Betacoronavirus* family that has high homology to SARS-CoV, caused an unprecedented CoV-related pandemic, and posed major threat to global health and stability^3,4^. SARS-CoV-2 infection, like other viral infections, triggers adaptive immune response, including generation of neutralizing antibodies^5^. In response to the pandemic, there is a pressing need for extensive serological testing to estimate the levels of COVID-19 antibodies in high-risk communities and to evaluate the effectiveness of SARS-CoV-2 vaccine in generating neutralizing antibodies. Hence, identification of a robust antigen and serological method is important to facilitate large-scale screening of COVID-19 antibodies. The SARS-CoV-2 S protein is a good candidate for antigen as many COVID-19 antibodies have been found to target the S protein^6–10^. In this regard, neutralizing antibodies against SARS-CoV-2 or the closely related SARS-CoV mainly target the RBD on the S1 subunit of the S protein (Fig. 1a), a region that engages the receptor, ACE2, on the host cell^11^. The full-length S protein, which consists of the S1 and S2 subunits, normally exists in a trimeric state with one of the three RBDs being in an accessible conformation^12,13^. Our aim is to determine which components or forms of the S protein is more sensitive and specific in ELISA assays to detect and differentiate anti-SARS-CoV-2-specific antibodies from antibodies elicited by the widely circulating CoVs.

**Fig. 1:**
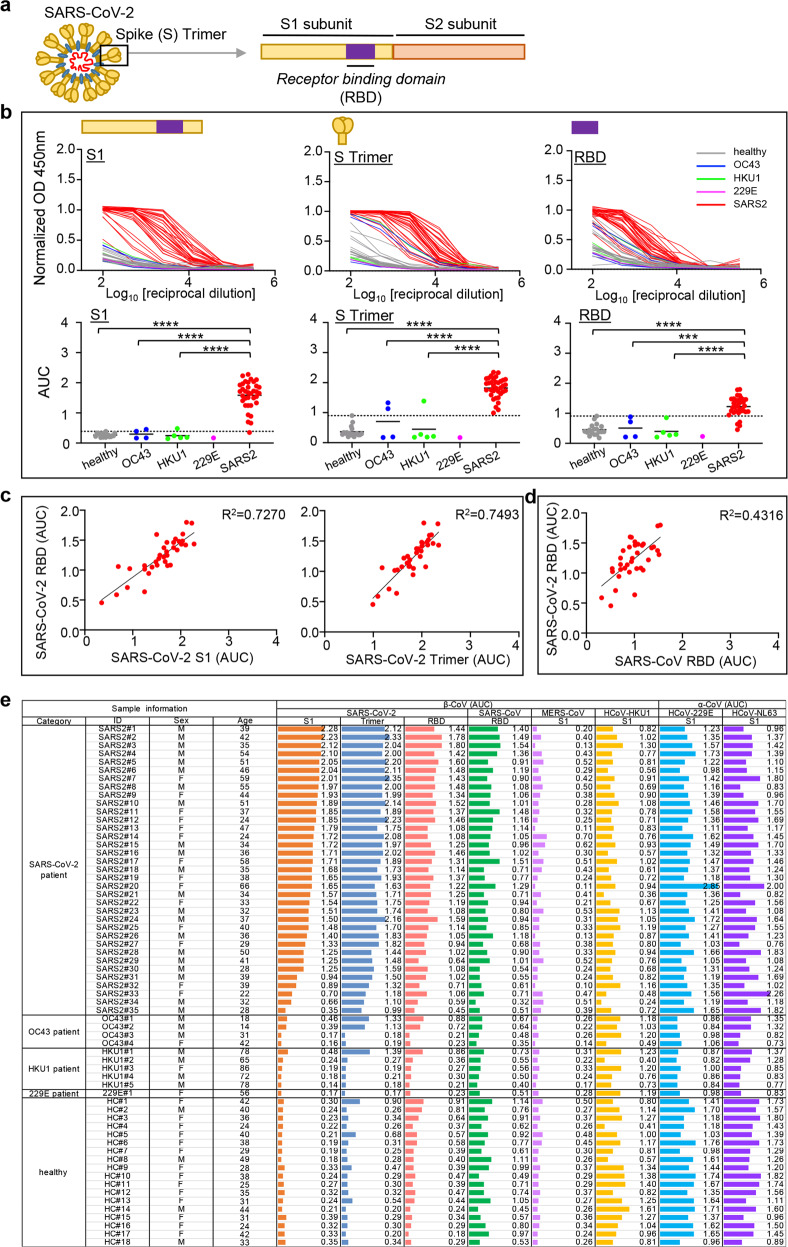
ELISA assays to detect anti-SARS-CoV-2 antibodies in human coronaviruses infected individuals. **a** Schematic showing SARS-CoV-2 S trimer, S1 and RBD. **b** ELISA measurement of plasma reactivity to RBD, S1, and S trimer. *Y*-axis is OD 450 nm normalized to the mean absorbance of CR3022 in each plate. *X*-axis is log reciprocal plasma dilution (upper row). Normalized area under the curve (AUC) of 18 controls (gray) and 45 patients (35 patients infected with SARS-CoV-2, red; 4 patients infected with HCoV-OC43, blue; 5 patients infected with HCoV-HKU1, green; and 1 patient infected with HCoV-229E, magenta) for IgG binding to SARS-CoV-2 S1, S trimer, and RBD (lower row). Data were expressed as mean. *P < 0.05, **P < 0.01, ***P < 0.001, ****P < 0.0001. **c** Correlation between AUC of anti-SARS-CoV-2 RBD and AUC of anti-SARS-CoV-2 S1 (left) or anti-SARS-CoV-2 S trimer (right). **d** Correlation between AUC of anti-SARS-CoV-2 RBD and AUC of anti-SARS-CoV RBD. **e** Demographic details and normalized AUC of all samples for IgG binding to β-CoV (SARS-CoV-2 S1, S trimer, RBD, SARS-CoV RBD, MERS-CoV S1, and HCoV-HKU1 S1) and α-CoV (HCoV-229E S1 and HCoV-NL63 S1) presented in bar chart.

We performed ELISA on plasma of 35 COVID-19 convalescent patients and 18 healthy controls, using SARS-CoV-2 RBD, S1, or S trimer antigens (Fig. 1b). As controls for normalization of OD measurements in different plates, we included purified CR3022, an antibody specific to SARS-CoV that also cross-binds to SARS-CoV-2^14^ in every plate (Supplementary Fig. S1a). We then calculated the area under the curve (AUC) of the normalized OD using two different methods, first, where the *X*-axis is the log of reciprocal dilution (Fig. 1b) and second, where the *X*-axis is reciprocal dilution (Supplementary Fig. S1b, c). The former method is better than the latter in discerning COVID-19 antibodies from convalescent patient and is used in subsequent analysis. In the former method, S1 and S trimer are both sensitive antigens for detecting COVID-19 antibodies, as all 35 COVID-19 patients had AUC levels above or close to the borderline of the healthy control threshold, which is determined by the highest AUC in the healthy control (Fig. 1b, bottom panel). In the RBD group, the AUC levels of four COVID-19 patients fall below the healthy control threshold. Since S1 and trimer contain other epitopes besides RBD, the sensitivity of these antigens over RBD may be due to their ability to capture non-RBD-binding antibodies. Nevertheless, S1 and S trimer mainly detect COVID-19 antibodies targeting the RBD, as we observed positive correlation between AUC levels measured by RBD and S1 or S trimer (Pearson correlation = 0.7270 and 0.7493), respectively (Fig. 1c). Therefore, by using an appropriate method of AUC calculation, we show that S1 and S trimer are better antigens than the RBD in terms of sensitivity against COVID-19 antibodies.

To determine whether the antigens that were tested cross-bind with antibodies elicited by other widely circulating CoVs, we also included samples from patients that were infected with either HCoV-OC43 (*n* = 4), HCoV-HKU1 (*n* = 5), or HCoV-229E (*n* = 1), which were collected prior to SARS-CoV-2 outbreak. For HCoV-HKU1 and HCoV-229E, we also performed ELISA with their corresponding S1 antigens. We observed varying degrees of binding activities to their own antigens, similar to the variation that was observed for binding activities of SARS-CoV-2-infected samples to SARS-CoV-2 antigens. Our analysis shows that S trimer may cross-bind at low levels with antibodies elicited by other circulating CoV strains, such as those from the *Betacoronavirus* family HCoV-OC43 and HCoV-HKU1 (Fig. 1b). This is perhaps due to higher homology of the S2 portion of the trimer between SARS-CoV-2 and HCoV-OC43 and HCoV-HKU1 compared to the S1 portion alone (Supplementary Fig. S1d). Thus, with respect to the specificity of the antigen toward COVID-19 antibodies, the S1 antigen surpasses the S trimer. Taken together, the S1 antigen performed better than the RBD, and S trimer in both sensitivity and specificity towards COVID-19 antibodies.

We also tested whether antibodies from COVID-19 patients cross-reacted with antigens from other CoV. We performed ELISA using plasma from the same patients with SARS-CoV RBD and S1 subunits of MERS-CoV, HCoV-HKU1, HCoV-229E, and HCoV-NL63. The latter two are circulating CoV from the *Alphacoronavirus* family. Our results show that SARS-CoV RBD which has 73.8–74.9% amino acid identity with SARS-CoV-2 RBD^4^ cross-binds with antibodies from COVID-19 patients, as there is a positive correlation (Pearson correlation = 0.4316) between AUC levels measured by SARS-CoV-2 RBD and SARS-CoV RBD (Fig. 1d). This is expected considering the similarity of the RBD sequence between these two strains and that antibodies elicited by SARS-CoV-infected patients can cross-neutralize SARS-CoV-2^8^. Antigens from the circulating human CoVs, including HCoV-HKU1, HCoV-229E, and HCoV-NL63 seemed to bind antibodies from COVID-19 patients, as well as healthy controls in varying degree, suggesting prior widespread infection of these CoVs in the general population (Fig. 1e and Supplementary Fig. S2a, b). S1 antigen from MERS-CoV, a virus from the *Betacorovirus* that caused severe disease and originated from the Middle East generally does not cross-react with antibodies in our cohort of COVID-19 patients and controls (Fig. 1e and Supplementary Fig. S2a). We have not observed positive correlation between AUC levels measured by S1 of SARS-CoV-2 and S1 antigen of other tested CoV strains, suggesting that the latter antigens are probably not specific to COVID-19-specific antibodies (Supplementary Fig. S3). However, it is possible that some COVID-19-specific antibodies in certain convalescent patients may cross-react with the circulating mild CoV strains. Further experiments would be required to isolate such cross-reactive antibodies.

Our results show that SARS-CoV-2 S1 is a robust antigen in serological assays to detect SARS-CoV-2-specific antibodies. S1, being more sensitive than RBD, and more specific than S trimer, is the optimal antigen among the three SARS-CoV-2 S antigens tested. Recently, SARS-CoV-2-neutralizing antibodies targeting the N terminal region (NTD) of S1 were isolated using the S trimer^15^, suggesting that the S trimer may be better in capturing COVID-19 antibodies. While the native state of S trimer may be in the prime conformation to capture all SARS-CoV-2-specific antibodies, including those that target regions outside the RBD and S1^15^, it also binds nonspecifically to antibodies elicited by other closely related circulating CoV strains. Thus, when considering both sensitivity and specificity criteria of the antigens in serological assays, S1 antigen is preferable over S trimer. Recent studies have shown that a two-step procedure using both SARS-CoV-2 RBD and S trimer antigens in immunoassays demonstrated high sensitivity and specificity for COVID-19 antibodies^16,17^. In this regard, our results show that S1 antigen alone can achieve high sensitivity and specificity for detecting COVID-19 antibodies, thereby increasing efficiency and reducing cost of the immunoassays. The ease of purification of S1 protein over the full-length S protein or the trimeric form also makes it favorable for large-scale serological testing. While the S1 antigen of SARS-CoV-2 is able to distinguish antibodies targeting SARS-CoV-2 from the widely circulating CoV, it may not be able to distinguish antibodies targeting SARS-CoV, considering the high similarity in the amino acid identity between these two strains (Supplementary Fig. S1d). In this case, further experiments using pseudovirus neutralization may be required when determining COVID-19 antibodies from a certain population that may have been previously exposed to SARS-CoV. Another caveat in this study is the relatively small sample size studied. Finally, we propose that S1 antigen is the optimal reagent in facilitating large-scale serological assays in the evaluation of various SARS-CoV-2 vaccines that are currently in development.

## Supplementary information

Supplementary Information

## Supplementary information

**Supplementary Information** accompanies the paper at (10.1038/s41421-020-00224-3).
