# Supplementary material for: Sensitivity and specificity of SARS-CoV-2 S1 subunit in COVID-19 serology assays
Source: Cell Discov. 2020 Oct 27;6:75. doi: 10.1038/s41421-020-00224-3 (PMC7589479; doi:10.1038/s41421-020-00224-3)
Supplement: Supplementary file 1 — Supplementary Information [file 41421_2020_224_MOESM1_ESM.pdf]

Supplementary Data S1

Materials and Methods:

Human specimen

This study included 35 adult COVID-19 convalescent patients from Shanghai Public Health Clinical Center. All COVID-19 patients were confirmed with symptomatic SARS-CoV-2 infection and positive for SARS-CoV-2 nucleic acid test. Blood samples were collected from these patients around two weeks after discharging from the hospital. Human CoV infected blood samples (HCoV-OC43; n=4, HCoV-HKU1; n=5, and HCoV-229E; n=1) were obtained from Ruijin Hospital, Shanghai between Dec 2018 to June 2019, and were confirmed by the corresponding nucleic acid testing and tested negative for SARS-CoV-2. All the patients had signed informed consents. The study was approved by Shanghai Ethics Committee for Clinical Research (SECCR/2020-04-01).

Purification of CR3022 monoclonal antibody

The SARS-CoV specific antibody CR3022 was produced in transiently transfected 293S cells. The amino acid sequence of heavy and light variable domain sequences were obtained from GenBank: ABA54613 and ABA54614. Antibody variable domain gene sequences were synthesized by Genewiz and cloned into human vector IgG2 and human kappa expression vectors. The heavy and light chain plasmids were co-transfected in 293S cells by PEI at a ratio of 1:1 and were purified using Protein G Sepharose and buffer exchanged into PBS before use. CR3022 antibody was analyzed via a standard SDS-PAGE gel to check protein integrity.

Mice immunization

Mice aged 8-12 weeks were immunized with immunogen mix (HCoV-229E S1, Cat# 40601-V08H, Sino Biological; MERS-CoV S1, Cat# 40069-V08H, Sino Biological; HCoV-HKU1 S1, Cat# 40602-V08H, Sino Biological) by injecting the inner thigh of the two rear legs of each mouse. Blood were collected at day 21 after the first

immunization and the serum were used as the positive control in the following ELISA assay.

ELISA

Human plasma samples were heat-inactivated in 56 °C for 30 minutes. 96-well plate was coated with SARS-CoV-2 antigens (RBD, Cat#DRA36, Novoprotein; S1, Cat# 40591-V08H, Sino Biological; trimeric spike, Cat# DRA49, Novoprotein), α-CoV antigens (SARS-CoV1 RBD, Cat# 40150-V08B2, Sino Biological; HCoV-HKU1 S1, MERS-CoV S1) and β-CoV antigens (HCoV- 229E S1, HCoV-NL63 S1, Cat# 40600-V08H, Sino Biological) (2 μg/ml) overnight at 4°C. Plates were washed 3 times with PBST (PBS with 0.05% Tween 20) and blocked with 2% BSA in PBST for 1 hour at room temperature. Plasma samples serially diluted in PBS were added and incubated for 2 hours at RT. After 5 times wash with PBST, a 1:3000 dilution of horseradish peroxidase (HRP)-conjugated goat anti-human (Cat# SA00001-17, proteintech) or anti-mouse IgG (Cat# SA00001-1, proteintech) antibody was added to each well for 1 hour at room temperature. The plates were then washed 4 times again and the TMB peroxidase Substrate were added and terminated the color reaction with 1M sulfuric acid. The absorbance at 450 nm (OD450) was recorded immediately. CR3022 or immunized mouse serum were set as positive control on each plate. All tests were performed in triplicate.

Sequence analysis and alignment

Sequence alignments of RBD, S1, S2, S proteins between SARS-CoV-2 and other coronaviruses were made with Clustal Omega. Sequences used were RefSeq NC_045512 (SARS-CoV-2), RefSeq NC_004718 (SARS-CoV), RefSeq NC_019843 (MERS-CoV), NC_006577 (HCoV-HKU1), RefSeq NC_006213 (HCoV-OC43), RefSeq NC_005831 (HCoV-NL63), and RefSeq NC_002645 (HCoV-229E).

Statistical analysis

Statistical analyses were carried out using GraphPad Prism 8.0. OD450 values were normalized to the mean of positive controls before further analysis. Data are indicated as mean and p values were calculated by One-way ANOVA followed by Dunnett’s multiple comparisons. Correlations were calculated using standard Pearson correlation. P values less than 0.05 were considered to be statistically significant. In the ELISA assay, area under the curve (AUC) is used as a cumulative measurement. The AUC calculations are equivalent to taking a weighted average of all the Y values with equally spaced X values. The area is still a weighted average of the responses when the X values are not equally spaced, but the weights account for the unequal spacing with the responses corresponding the widely spaced doses having more weight than responses from doses that are more closely spaced. Two different methods in AUC calculations were compared, where the X axis is log [reciprocal dilution] or where the X axis is reciprocal dilution.

Supplementary Fig. S1

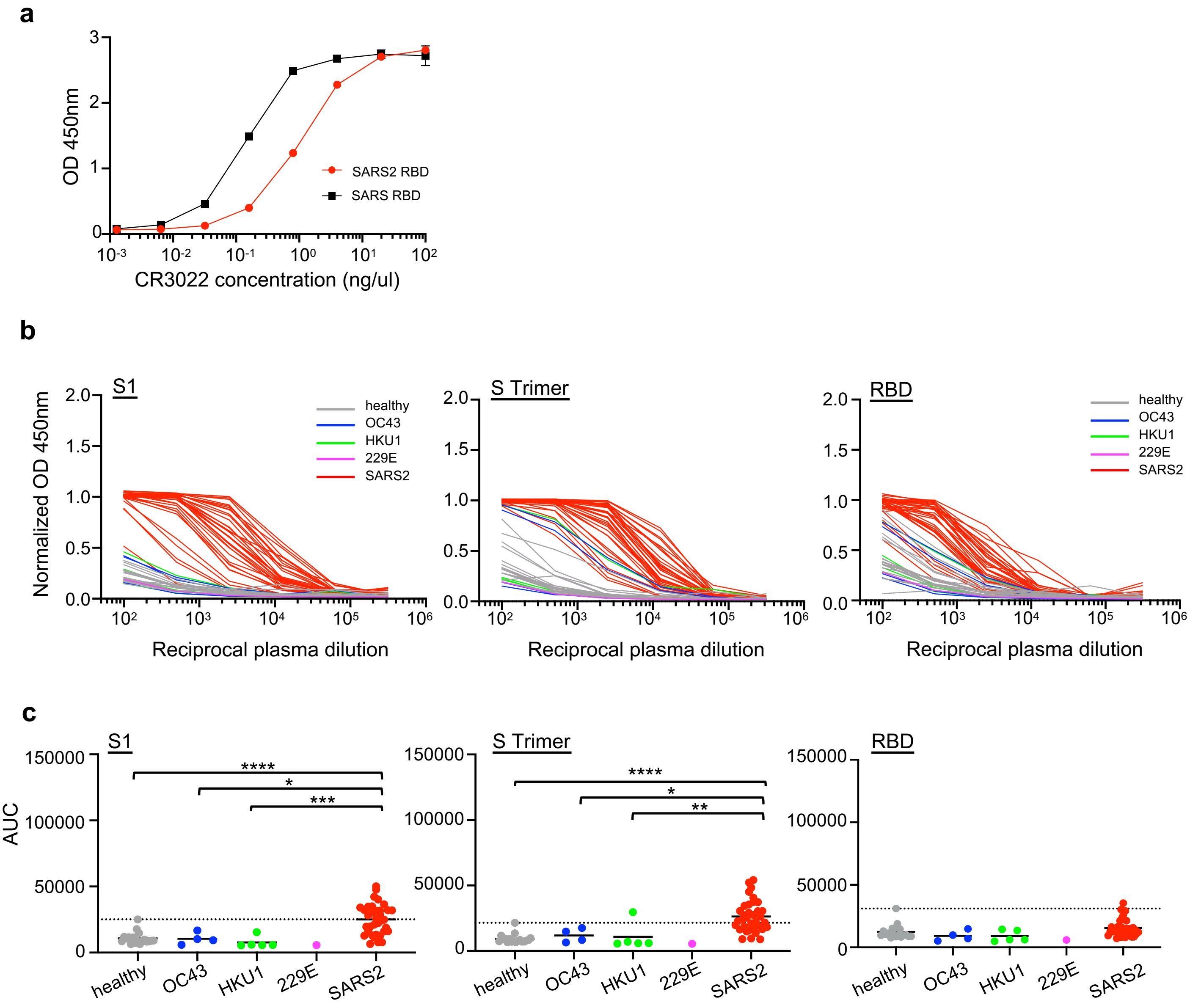

d

Genus	CoVs	% of amino acid identity to SARS-CoV-2			
		RBD	S1	S2	S
β	SARS-CoV	74.2	65	88.7	76.4
	MERS-CoV	20.6	19.7	39.6	29.8
	HCoV-HKU1	15.5	19.5	26.9	28.5
	HCoV-OC43	16.2	20.2	28.4	29.3
α	HCoV-NL63	10.8	15.4	31.9	23.3
	HCoV-229E	8.1	18.3	32.6	25.7

Supplementary Fig. S1 Characterization of CR3022 and Analysis of AUC where X axis is Reciprocal Dilution. a Purified CR3022 antibody show cross-binding to SARS-CoV and SARS-CoV-2 RBD; **b** ELISA results from the same experiments as Figure 1b were presented by reciprocal dilution of samples (X axis) plotted against normalized OD450nm value (Y axis); **c** Normalized AUC for IgG binding to SARS-CoV-2 antigens (Y axis) were calculated by reciprocal dilution of samples (X axis). Data were expressed as mean. **p*< 0.05, ***p*< 0.01, ****p*< 0.001, *****p*< 0.0001; **d** Sequence homology analysis of SARS-CoV-2 RBD, S1, S2 and full-length S compared to that of other α- and β-CoVs.

Supplementary Fig. S2

a β-CoV

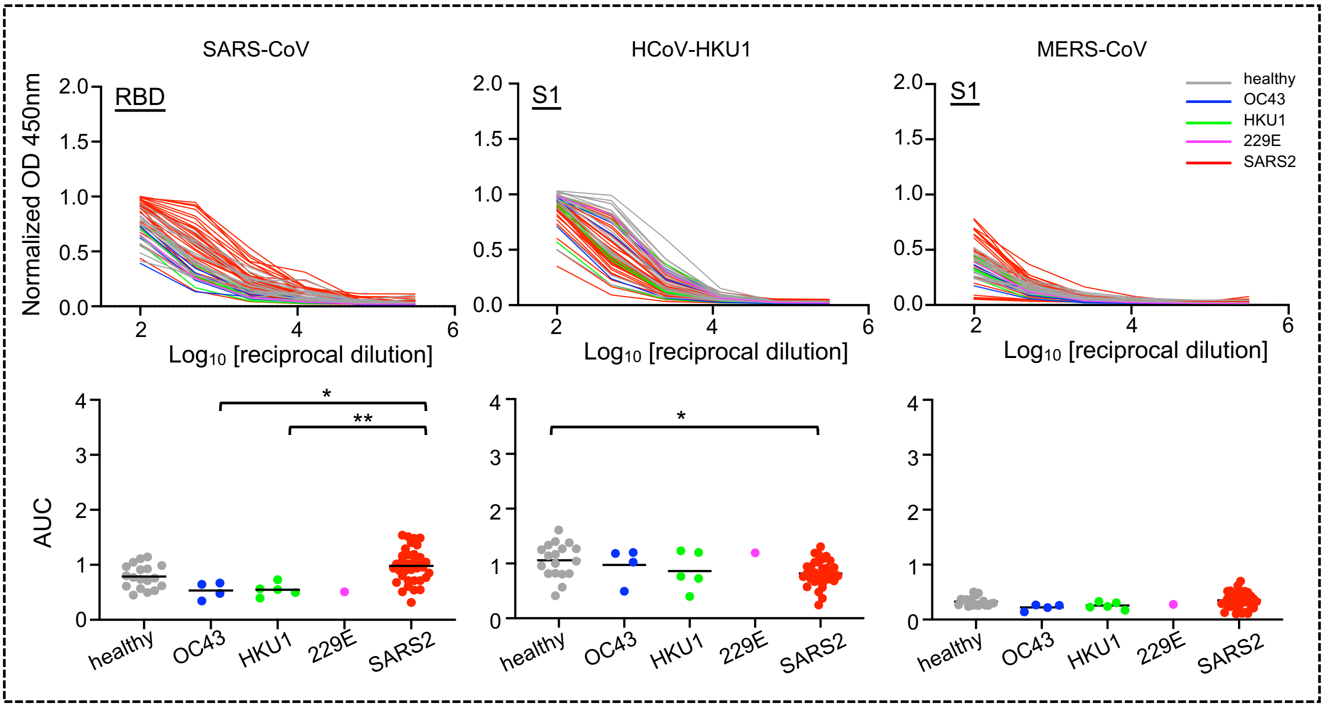

b α-CoV

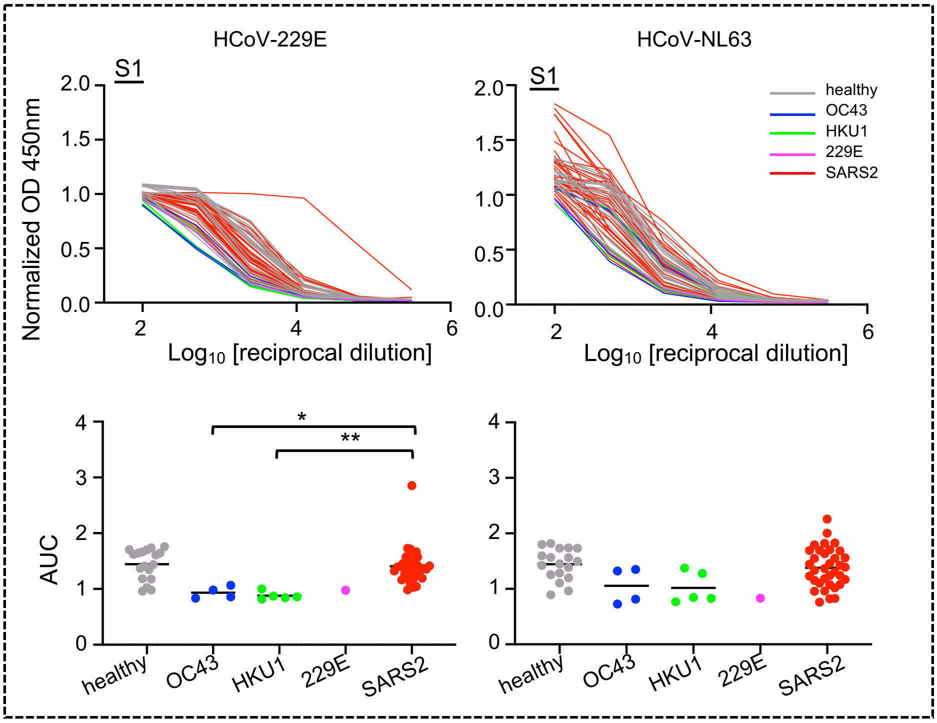

Supplementary Fig. S2 Detection of β-CoV and α-CoV-Specific Antibodies in Human Coronaviruses Infected Individuals by ELISA assay. a ELISA measurement of plasma reactivity to β-CoV (SARS-CoV, MERS-CoV, HCoV-HKU1) (upper row). Normalized AUC of 18 controls and 45 patients for IgG binding to β-CoV (SARS-CoV, MERS-CoV, HCoV-HKU1) calculated by log [reciprocal dilution] (X axis); **b** ELISA measurement of plasma reactivity to α-CoV (HCoV-229E, HCoV-NL63). Normalized AUC of 18 controls and 45 patients for IgG binding to α-CoV (HCoV-229E, HCoV-NL63) calculated by log [reciprocal dilution] (X axis). Data were expressed as mean. *$p < 0.05$, **$p < 0.01$, ***$p < 0.001$, ****$p < 0.0001$.

Supplementary Fig. S3

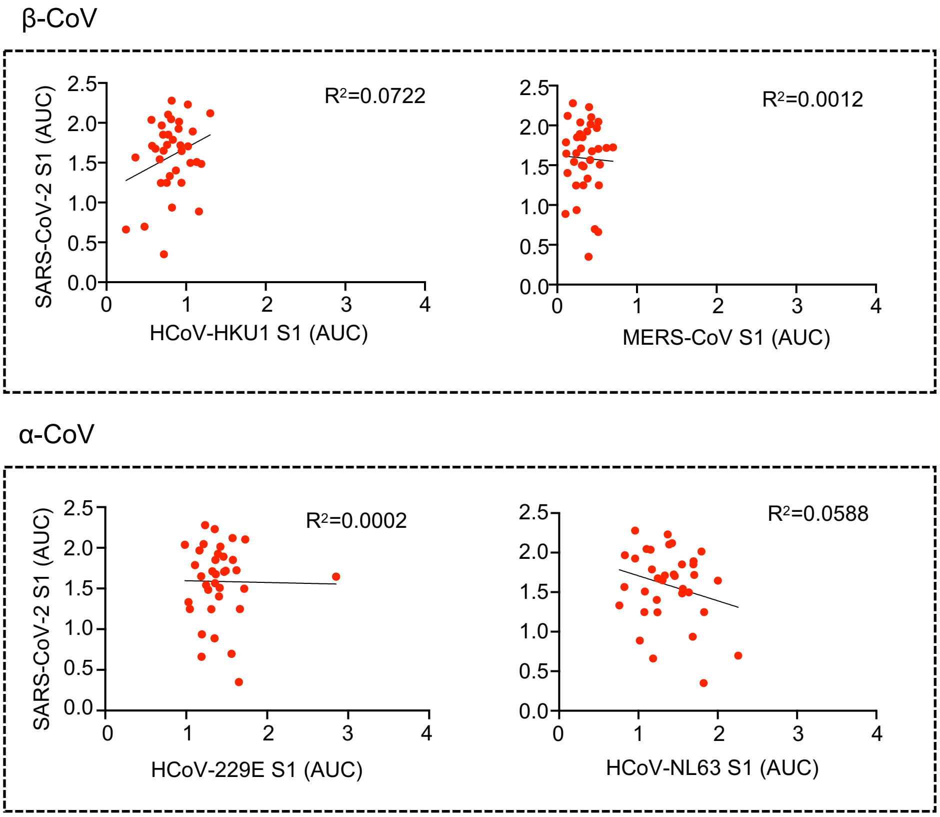

Supplementary Fig. S3. Correlation between AUC of anti-SARS-CoV-2 S1 and AUC of other anti-β-CoV and anti-α-CoV IgG. Normalized AUC of 35 COVID-19 patients calculated by log [reciprocal dilution] (X axis).
